# Partial Fractionation of Venoms from Two Iranian Vipers, Echis carinatus and Cerastes persicus Fieldi and Evaluation of Their Antiplatelet Activity 

**Published:** 2012

**Authors:** Toktam Mehdizadeh Kashani, Hossein Vatanpour, Hossein Zolfagharian, Hasan Hooshdar Tehrani, Mohammad Hossein Heydari, Farzad Kobarfard

**Affiliations:** a *Department of Toxicology, School of Pharmacy, Shahid Beheshti University of Medical Sciences, Tehran, Iran. *; b *Pharmaceutical Sciences Research Center, Shahid Beheshti University of Medical Sciences, Tehran, Iran. *; c *Department of Venomous Animals and Antivenom Production, Razi Vaccine and Serum Research Institute, Karaj, Iran. *; d *Department of Medicinal Chemistry, School of Pharmacy, Shahid Beheshti University of Medical Sciences, Tehran, Iran. *; e *Proteomics Research Center, Paramedical School, Shahid Beheshti University of Medical Sciences, Tehran, Iran. *; f *Phytochemistry Research Center, Shahid Beheshti University of Medical Sciences, Tehran, Iran. *

**Keywords:** Anticoagulant, Antiplatelet aggregation, Snake venom, *Echis carinatus*, *Cerastes persicus fieldi*, Gel filtration

## Abstract

Platelet aggregation inhibitory effect and anticoagulant properties of fractions separated from the venoms of *Cerastes persicus fieldi *and *Echis carinatus *were investigated.

The partial fractionation was performed on a Sephadex G-100 column. Two fractions separated from *Cerastes persicus fieldi *showed anti platelet aggregation activity on ADP (200 μM)-induced platelet aggregation (ca 80% inhibition). Attempts to measure the antiplatelet aggregation activity of crude *Echis carinatus *venom and its fractions were not successful due to the protein coagulation of the plasma samples after the addition of venom. Anticoagulant activities of venoms were also evaluated. Total venom of *Echis carinatus *showed anti coagulant activity in PT test, while its fractions showed procoagulant activity.

## Introduction

Snake venoms are complex mixtures of pharmacologically active proteins and polypeptides. They play an important role in incapacitating and immobilizing, as well as in digesting prey (1, 2). Thus toxins have evolved to specifically target various critical points in the physiological systems of prey animals. Neuromuscular and circulatory systems are the two main physiological systems that are targeted by a great many toxins, as interruption(s) in these systems make the prey succumb to the venom in a short time. Nerve toxins are generally found in the Hydrophidae and Elapidae venoms whereas hemorrhagic and myonecrotic toxins are generally found in the venoms of the Viperidae and Crotalidae families of snakes. Over the years, a number of toxins that affect blood circulation have been isolated and characterized from various snake venoms (3-7). Some of them affect platelet aggregation (for recent reviews, see references 8-10), whereas others affect blood coagulation. Venom proteins affecting blood coagulation can functionally be classified as pro- coagulant or anticoagulant proteins on the basis of their ability to shorten or prolong the blood-clotting process. Pro-coagulant proteins are either serine proteinases or metalloproteinases. Their sizes vary between 24 kDa and 300 kDa. They induce blood coagulation either by specifically activating zymogen, one of the blood coagulation factors, or by directly converting soluble fibrinogen into an insoluble fibrin clot. Structural and functional details of these pro-coagulant proteins from snake venoms have been recently reviewed (11-14). Snake venom toxins that prolong blood coagulation are proteins or glycoproteins with molecular masses ranging from 6kDa to 350 kDa. These factors inhibit blood coagulation by different mechanisms. Some of these anticoagulant proteins exhibit enzymatic activities, such as PLA2 (phospholipase A2) and proteinase, whereas others do not exhibit any enzymatic activity (3-8, 11). Aberration in normal blood coagulation functions can result in thrombotic disorders or haemorrhage. In thrombosis, largely unknown conditions promote the apparently spontaneous formation of clots large enough to block circulation. Formation of such blocks in the arteries supplying vital organs, such as the heart or brain, can cause myocardial infarction or stroke respectively. Thus a life-saving mechanism of blood coagulation becomes a potentially life-threatening disease mechanism. Several conditions, such as atherosclerosis, contribute significantly to promote the spontaneous initiation of clotting. Anticoagulants are pivotal for prevention and treatment of thromboembolic disorders, and approximately 0.7% of the western population receives oral anticoagulant treatment (15). With the increasing aging population throughout the world, more people will require antithrombotic therapies in the future. Thus various new anticoagulant and antiplatelet agents are being sought after. Proteins from snake venom affecting blood coagulation and platelet aggregation can provide us with new lead compounds to design novel therapeutic agents, providing new paradigms in the treatment of thromboembolic disorders (16).

Viperidae venoms mainly cause hemorrhaging and coagulation disorders and as such provide a rich source of pharmacologically-active proteins and peptides for studying the clotting cascade as well as platelet glycoprotein receptors (17).

In the present study two snakes, *Echis carinatus *and *Cerastes persicus fieldi *from viperidae family were chosen and their venoms were subjected to fractionation processes. The separate fractions were examined for their effect on blood coagulation and ADP-induced platelet aggregation.

## Experimental

Fresh crude venoms from *Echis carinatus *and *Cerastes persicus fieldi *were obtained directly from local snakes in Iran, lyophilized and stored at 4 °C in dark glass bottles before use.

The average length of the snakes was 50 cm with an approximate age of 30 months.

Sephadex G-25, -50 and -100 were purchased from Pharmacia, Sweden.

ADP reagent was purchased from Hart Biological Co., UK.

Calcium chloride was purchased from Baharafshan Institute, Iran.

Activated partial thromboplastin time (APTT) reagent and prothrombine time reagent (Thromboplastin-D) were obtained from Fisher Scientific Co., U.S.A.

All of the other reagents were of analytical grade available from commercial sources.

Aggregation was measured on an APACT 4004 aggregometer (LABitec, Arensburg, Germany).

Clotting times were recorded using an opto-mechanical coagulation analyzer (Coa DATA 501, LABitec, Germany).

Protein concentration was determined spectrophotometrically at 220 nm, 260 nm and 280 nm by using UV-160A recording spectrophotometer (Shimadzu, Japan).

*Methods*

Gel filtration : About 15 mg of the crude venom of *E. Carinatus *was dissolved in 2 mL Tris buffer (pH 8, 0.1 M), and loaded on a Sephadex G-100 column, previously equilibrated with the Tris buffer and then eluted with the same buffer. Attempts to use Sephadex G-25 and G-50 did not give good separation. Fractions were collected in 2 mL portions at a flow rate of 60 mL/h. In the initial Sephadex G-100 fractionation of the crude *Echis Carinatus *venom, 5 fractions were obtained by monitoring the columns eluates at 280 nm, 260 nm and 220 nm. 

In another attempt crude venom of *Cerastes Persicus Fieldi *(30 mg) was applied to Sephadex G-100 column using Tris buffer (0.1 M) and pH 8. In the initial Sephadex G-100 fractionation of the crude *Cerastes Persicus Fieldi *venom 4 fractions were obtained by monitoring the columns eluates at 280 nm, 260 nm and 220 nm. 

*Platelet aggregation*

Platelet aggregation was determined according to born method (18). Briefly, blood samples were collected from healthy human donors and mixed with 3.8% sodium citrate (9:1 v/v). Citrated blood was immediately centrifuged for 8 min at 1000 rpm at room temperature. After removal of platelet-rich plasma (PRP) thus obtained, the remaining blood was recentrifuged at 3000 rpm for an additional 15 min and the platelet-poor plasma (PPP) such obtained was mixed with PRP to give a platelet count of about 250,000/mm3. One microliter of the solution containing the toxin was added to 500 μL PRP, three minutes before initiation of the aggregation by addition of 5 μL ADP. 

The maximum level of platelet aggregation after the addition of 5 μL ADP was quantified on a four-channel light transmission aggregometer. The measurements were performed in triplicates and within 2 h from blood sampling. 

*Blood coagulation*

Prothrombin time (PT Assay) : Prewarmed platelet poor plasma (PPP, 45 μL) and sample aliquots (5 μL) were mixed for 300 sec and 100 μL of prewarmed thromboplastin-D was then added and clotting time was recorded. 

Partial thromboplastin time (PTT): Prewarmed platelet poor plasma (45 μL) , sample aliquots (5 μL) and prewarmed APTT reagent (50 μL) were incubated for 300 sec and 50 μL of 0.025 M calcium chloride was then added and clotting time was recorded. 

Protein determination: Protein determination was measured by the method of lowry *et al*. (19), using bovine serum albumin (BSA) as standard. 

## Results

*Cerastes persicus fieldi*

**Figure 1:**
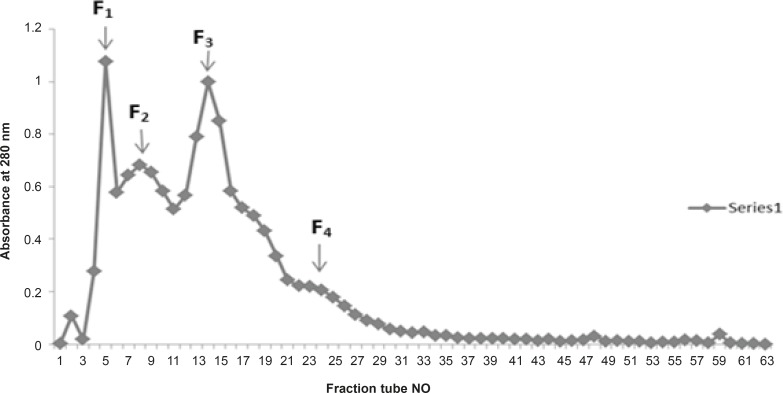
Sephadex G-100 chromatography of Iranian *Cerastes persicus fieldi *venom

Fractionation of *Cerastes persicus fieldi *by gel filtration on Sephadex G-100 yielded 4 major fractions F1-F4 (Figure 1). 

**Figure 2:**
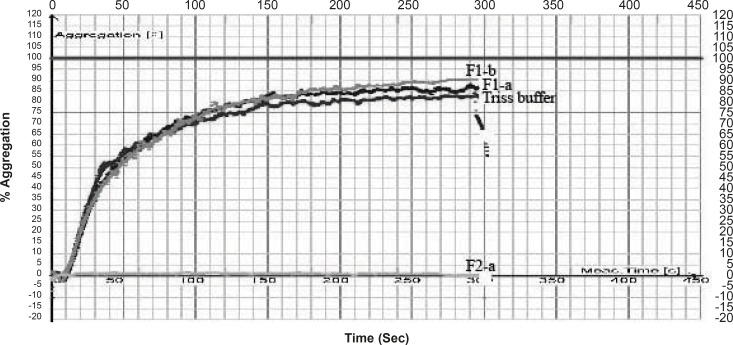
Anti platelet effect of fractions separated from Cerastes persicus fieldi venom on platelet aggregation induced by ADP (200 μM).

**Figure 3:**
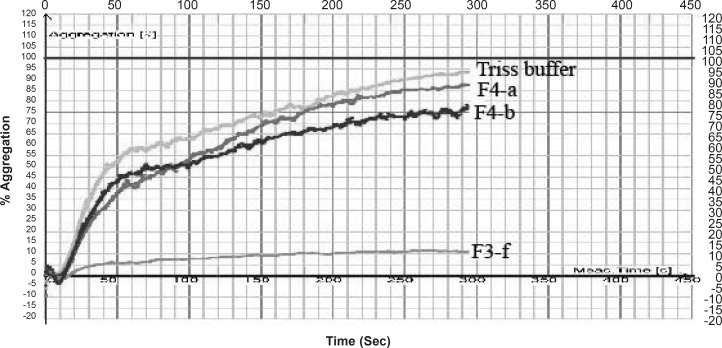
Anti platelet effect of fractions separated from Cerastes persicus fieldi venom on platelet aggregation induced by ADP (200 μM).

In case of *Cerastes persicus fieldi *Fraction F2-a and F3-f possessed the most potent inhibitory activity on ADP (200 μM)-induced platelet aggregation (Figure 2, 3).

The inhibitory activity of F2-a was 61 times higher than that of the control (buffer) (Table 1) .

Fraction F3-f decreased the % aggregation induced by ADP, from 95% ( for control) to 11.42% (Table 1). Crude venom of *Cerastes persicus fieldi *and its fractions were evaluated for PT and PTT tests. Neither the total venom nor its fractions showed anticoagulant or procoagulant activity.

**Table 1:** Anti platelet activity of fractions separated from Cerastes persicus fieldi venom

Fraction	Maximum aggregation (%)
F1-a	86.90
F1-b	90.31
F2-a	1.35
F2-b	26.77
F2-c	31.97
F2-d	40.58
F3-a	15.58
F3-b	21.13
F3-c	35.56
F3-d	48.35
F3-e	44.85
F3-f	11.42
F4-a	87.74
F4-b	77.66
F4-c	62.48
F4-d	54.49
F4-e	38.54
F4-f	69.01
Total venom (200 μg/mL)	33.12

*Echis carinatus*

**Figure 4:**
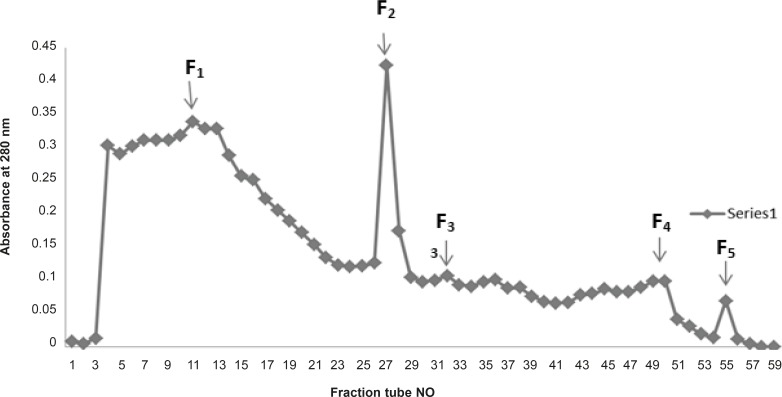
Sephadex G-100 chromatography of Iranian Echis carinatus venom

In another attempt fractionation of crude *Echis carinatus *venom by gel chromatography on a column of Sephadex G-100 yielded 5 major fractions F1-F5 (Figure 4).

Measurement of anti-platelet aggregation activity for the total venom of *Echis carinatus *and its fractions were not successful due to the coagulation of the plasma even after several steps of diluting the venom. Crude venom from *Echis carinatus *was assayed for PT and PTT tests. The venom (200 μg/mL) showed anticoagulant activity in PT test (87.6 s) (Table 2). In the initial Sephadex G-100 fractionation of the crude *Echis carinatus *venom, five major fractions were obtained as shown in Figure 4. All the fractions were tested for their anticoagulation activity and all fractions showed procoagulant effect.

**Table 2:** Anticoagulant activity of Echis carinatus total venom

**Sample**	**PT**	**INR**	**Ratio**
Total venom (200μg/mL)	87.6 s	10	6.8%
Plasma without buffer and venom	15.8 s	1.92	45.5%
Buffer	13.5 s	1.45	64.9%

## Discussion

*Cerastes persicus fieldi*

We isolated two potent platelet aggregation inhibitors from *Cerastes persicus fieldi *by gel filtration on sephadex G-100 column chromatography. Two fractions contained components that possess inhibitory activity on ADP-induced platelet aggregation (Figure 2 and 3).

Anticoagulants are pivotal for the prevention and treatment of thromboembolic disorders, and approximately 0.7% of the western population receives oral anticoagulant treatment (16). 

With the increasingly aging population throughout the world, more people will require antithrombotic therapies in the future. Thus various new anticoagulant and antiplatelet agents are being sought after. Proteins from snake venom affecting blood coagulation and platelet aggregation can provide us with new lead compounds to design novel therapeutic agents, providing new paradigms in the treatment of thrombotic disorders. Further complementary studies are needed to separate and characterize the components of this venom and determine the mechanisms of action of newly isolated anti-platelet aggregation proteins in the present study.

*Echis carinatus*

Attempts to measure the antiplatelet activity of the crude venom and its fractions were not successful due to the protein coagulation of the plasma samples, immediately after the addition of venom.

On the other hand, all the fractions showed procoagulant activity in PT and PTT assays while the crude venom surprisingly showed anticoagulant activity in PT test (87.6 s).

Snake venoms, particularly from the families crotalidae and viperidae, are complex mixtures of numerous molecules that can possess both procoagulant and anticoagulant properties (20).

Dambisya *et al*., (21) have reported that the different concentrations of *Calloselasma rhodostoma *(Malayan pit viper) venom showed the dual effect. Several procoagulant proteins from snake venoms have been isolated and characterized. They are either serine proteinases or metalloproteinases which activate specific zymogens of coagulation factors and initiate the coagulation cascade. These procoagulant proteins are useful in treating various thrombotic and hemostatic conditions and contribute to our understanding of molecular details in the activation of specific coagulation factors (12).

The clinical profile of *Echis carinatus *envenoming is usually reported as swelling, tissue necrosis and bleeding (22, 23). Systemic complications are primarily related to bleeding due to blood depleted of fibrinogen and factors V, VII, II and XIII. This is manifested as hemorrhage in different parts of the body including the gums, nasopharynx, gastrointestinal tract, urinary tract and central nervous system. The crude venom of Iranian *Echis carinatus *studied in the present study reemphasize the above-mentioned characteristics of this venom.

Procoagulant activity of the fractions isolated from this venom, on the other hand, is not consistent with the common clinical profile observed after envenomation by crude venom. Factor X activators are common in viperidae snake venoms and this study as well as others, suggest that snake venoms with procoagulant activity could contain factor X activators (24).

An interesting case report by Jinkins *et al*. indicates an arterial thrombotic complication in a 13-year old female which has led to cerebral infarction (23). Since the thromboses have occurred in a site distant from the site of viper bite, it could be due to a low grade disseminated intravascular coagulation caused by procoagulant components of *Echis carinatus *venom.

Further detailed mechanistic studies are needed for each component of *Echis carinatus *venom to decipher the diverse clinical hematological manifestations reported after envenomation. 
